# Is promoting euthanasia or assisted suicide really a good idea?

**DOI:** 10.4102/safp.v66i1.5948

**Published:** 2024-05-08

**Authors:** Allan Donkin

**Affiliations:** 1 Private Practice, Cape Town, South Africa

To the Editor,

There has been a recent spree of South African medical journal publications^1,2^ and news articles^3^ promoting the idea of legalising euthanasia in South Africa (SA), as DignitySA is engaging media attention in preparation for their upcoming court case, again attempting to legalise euthanasia in SA using the courts. The suicide of a South African, Carol de Swardt, was encouraged and assisted in Switzerland earlier in 2024.^3^

The facts of Carol’s story give us reason not to be too hasty to legalise euthanasia in SA and reveal how real help for people becomes undermined by euthanasia. Carol did not have a terminal illness but rather was living with a type of disability that is not insurmountable and is shared by many South Africans. Rather than being assisted in getting a mental health assessment for depression or suicidality, her self-report of wish-to-die was simply taken on face value as she was ushered towards her death. She had recently received over a R4 million pay-out for medical negligence, which has to be considered as a possible motivating factor for her family supporting her suicide.

As doctors, when someone presents with suicidal ideation, we have a responsibility to assist them in many ways, including sometimes even admission to hospital to prevent suicide. However, when euthanasia is legalised, a person’s wish to die cannot be restricted ultimately – as laws shift from having ‘safeguards’ (e.g., restricted to terminal care) to being allowed for anyone. There is no legal reason that can be sustained that can justify allowing autonomy for some people and not for others. This puts the most vulnerable people with the most compromised autonomy at the most risk:

Is this not an obvious pitfall of legalising euthanasia? How can anyone claim that people would not end the lives of family members for the sake of money, or that elderly people would not ask for or acquiesce to euthanasia out of a sense of guilt that they would otherwise be a burden on their families’ emotions and finances?^4^

… an expression of a wish to die is often more a question than a statement. ‘Am I of any worth?’ ‘Am I in the way?’ Patients are looking for the picture of themselves in the eyes of their beholders. If the answer is returned that they should be helped to die, it is an affirmation of what they suspected – that others now see them as worthless.^5^

The press has a responsibility to be restrained when reporting about suicide to protect against further suicides from a contagion effect through mimicry. However, the *YOU* article^3^ about Carol looks like an advert for international suicide trade, naming the organisation that can supply it as well as the price that can be paid for it.

There has been some editorial inconsistency in the publishing of articles about euthanasia in South African medical journals. In 2021, the editorial team at the *South African Medical Journal (SAMJ)* declined the publication of a scientific letter, ‘Physician-assisted suicide and euthanasia – who are the vulnerable?’,^5^ which cautioned against euthanasia – citing the reason as being that the topic of euthanasia is not appropriate for the *SAMJ* and should rather be submitted to the *South African Journal of Bioethics and Law* (*SAJBL*). The *SAJBL* did publish this scientific letter.^5^ However, the *SAMJ* has now readily published two editorials in one issue that promote euthanasia,^1,2^ and two of the six authors of the one editorial^1^ are emeritus editors of *SAMJ*.

The topic of euthanasia has been thoroughly debated in South African medical journals,^1,2,4,5,6,7,8,9^ and the editorial referenced did not introduce any new arguments or information. The assertion that palliative sedation (where the underlying illness causes the death) is analogous to euthanasia (where death is artificially introduced) has been refuted and is not the standard view in palliative care.^10^

Personal motivating factors are common, as opinions are formed on this subject. The suffering of dementia was mentioned in one of the *SAMJ* editorials.^2^ I have noticed in general practice that when patients develop dementia, it can be a devastating experience for loved ones as they move through stages of mourning, as the person they know and love is in a sense ‘no longer there’. However, it is often not the patient themselves that suffers (they may be quite comfortable and well cared for). It is not infrequent that people going through this mourning suggest that euthanasia should be a solution. But would it really be the human and moral solution to kill our citizens who are living with dementia? Would this really solve the existential problem of human frailty and suffering with which we are wrestling?

We are fortunate that our Constitution protects the human dignity (value) of the most vulnerable and also balances the very relative right to autonomy with safety for those most vulnerable whose autonomy is the most compromised: ‘the right to freedom *and security* of the person’.^11^

Hopefully, medical journals in South Africa will allow the publication of alternative views in order to facilitate a balanced discussion around legalisation of euthanasia.


Dr Allan Donkin General practitioner, palliative care doctor, Somerset West, South Africa (member of PalPrac, Association of Palliative Care Providers in Southern Africa).

